# Gestational Weight Gain Growth Charts Adapted to Japanese Pregnancies Using a Bayesian Approach in a Longitudinal Study: The Japan Environment and Children’s Study

**DOI:** 10.2188/jea.JE20210049

**Published:** 2023-05-05

**Authors:** Naho Morisaki, Aurélie Piedvache, Seiichi Morokuma, Kazushige Nakahara, Masanobu Ogawa, Kiyoko Kato, Masafumi Sanefuji, Eiji Shibata, Mayumi Tsuji, Masayuki Shimono, Toshihiro Kawamoto, Shouichi Ohga, Koichi Kusuhara, the Japan Environment and Children’s Study Group

**Affiliations:** 1 Department of Social Medicine, National Research Institute for Child Health and Development, Tokyo, Japan; 2 Department of Health Science, Graduate School of Medical Science, Kyushu University, Fukuoka, Japan; 3 Research Center for Environmental and Developmental Medical Sciences, Kyushu University, Fukuoka, Japan; 4 Department of Obstetrics and Gynecology, Graduate School of Medical Sciences, Kyushu University, Fukuoka, Japan; 5 Department of Pediatrics, Graduate School of Medical Sciences, Kyushu University, Fukuoka, Japan; 6 Japan Environment and Children’s Study, UOEH Subunit Center, University of Occupational and Environmental Health, Fukuoka, Japan; 7 Department of Obstetrics and Gynecology, School of Medicine, University of Occupational and Environmental Health, Fukuoka, Japan; 8 Department of Environmental Health, School of Medicine, University of Occupational and Environmental Health, Fukuoka, Japan; 9 Department of Pediatrics, School of Medicine, University of Occupational and Environmental Health, Fukuoka, Japan

**Keywords:** Bayesian, curves, charts, pregnancy weight, gestational weight gain

## Abstract

**Background:**

Tracking gestational weight gain (GWG) during pregnancy makes it possible to optimize pregnancy outcomes, and GWG growth curves are well suitable for this purpose. The GWG guidelines for Japanese were revised in 2021. However, currently, there are no GWG growth curves to guide women on how to gain weight to meet these guidelines.

**Methods:**

Using data on 96,631 live births from the Japan Environment and Children’s Study (JECS), we created descriptive GWG percentile curves estimating the trajectory of GWG required to meet the GWG guidelines stratified by pre-pregnancy body mass index (BMI). For both analyses, Bayesian mixed models with restricted cubic splines adjusted for maternal characteristics were used.

**Results:**

GWG curves substantially differed by pre-pregnancy BMI and were higher among multiparas and those with lower maternal age and with no previous disease. We estimated that underweight, normal weight, overweight, and obese women who gain 8.4 to 11.1 kg, 6.4 to 9.1 kg, 3.8 to 6.5 kg, and <1.9 kg at 30 weeks of gestation are on the trajectory to reach the new guidelines at 40 weeks of gestation.

**Conclusion:**

We provide GWG percentiles curves for Japanese women, as well as GWG trajectory curves to meet the new GWG recommendations. These results may help pregnant women monitor weight during pregnancy.

## INTRODUCTION

Gestational weight gain (GWG) is known to influence birth outcomes, including birth weight and risk of preterm and cesarean delivery.^1^^–^^3^ As both inadequate and excessive weight gain are known to increase risk of adverse outcomes, guidelines and recommendations have been created to inform women and practitioners how much weight should be gained during pregnancy.^1^^,^^4^ Recently, in March 2021, the Japanese Society of Obstetrics and Gynecology (JSOG) revised its GWG guidelines, which were adapted also by the Japanese Ministry of Health, Labour and Welfare. In these new guidelines, they advise women to gain 12 to 15 kg (versus 9 to 12 kg previously), 10 to 13 kg (versus 7 to 12 kg), 7 to 10 kg (versus no official recommendation) and ≤5 kg (versus no official recommendation) by 40 weeks in pre-pregnancy underweight, normal weight, overweight, and obese populations, respectively. However, these ranges do not suggest whether women are on track or gaining too fast or too slow, and may not be very informative to monitor or to conduct interventions on weight gain during pregnancy.

Fetal and child growth is usually monitored via growth charts, and there have been attempts to create GWG growth curves in the same way. Unfortunately, the methodological qualities of studies older than 2014 have been questioned in a systematic review by Ohadike in 2016.^5^ More recent studies published in the last decade have used frequentist multilevel models accounting for repeated measurements, with either a restricted cubic spline model or second degree fractional polynomial model for gestational age.^6^ The largest study to date was conducted by Santos et al^7^ from multiple cohorts in Europe, the United States, and Oceania. The study used generalized additive models for location, scale, and shape (GAMLSS) with a Box-Cox t distribution,^8^^,^^9^ a method which is nowadays considered the main method for making growth curves. However, its limitation is that it ignores dependence between observations.

Compared to these models, Bayesian mixed models may be able to create more accurate curves, as the method allows the number of measurements and the timing of measurements to vary by individual, allows inclusion of covariates that affect growth in the model, and can overcome power issues due to small sample size,^8^ in addition to incorporating non-independence of measures of each individual as in other multilevel models. While Bayesian mixed models have such strengths, their use remains rare; this has to our knowledge only been applied to construct one fetal growth chart^9^ and one GWG growth chart.^10^

Thus, in this study, we aimed to create GWG curves showing the distribution of weight gain among Japanese women using a recent national birth cohort, and from these curves, to suggest GWG trajectory curves to meet the new GWG recommendations.

## METHODS

### Study population

The Japan Environment and Children’s Study (JECS) is a nationwide birth cohort. Detailed methodology has been previously reported.^11^^,^^12^ In brief, pregnant women were recruited through the first antenatal visit at participating co-operating health care providers, as well as through the local government offices issuing the Mother-Child Health Handbook, from January 2011 until March 2014 in 15 Regional Centers throughout Japan. During pregnancy, participating women were asked to fill out two questionnaires, which captured demographics, lifestyle, and behaviors, as well as medical history; one questionnaire was administered at recruitment, and another one was administered at mid/late pregnancy. Birth characteristics and medical information were transcribed separately from medical records. The Review Board on epidemiological studies of the Ministry of the Environment as well as the ethics committees of all the participating institutions approved the JECS protocol. We obtained written informed consent from all participants.

In total, 103,099 pregnancies were registered. For this study, we used the dataset of the birth characteristics “jecs-ag-20160424”, which was released in June 2016 and revised in October 2016. We excluded stillbirths, deliveries with unknown gestational age at term, and those with less than three valid weight measures collected during antenatal visits, leaving 96,631 pregnancies ending in live births as our sample. Among these, we defined a low-risk population of 17,950 singleton pregnancies that ended in spontaneous vaginal delivery and had no evident complications during pregnancy or 1 month post-birth. The low-risk population excluded complicated pregnancies, as weight management were likely to be controlled by practitioners, and also excluded and those ending with induced deliveries, vacuum or forceps delivery, cesarean delivery, or unknown mode of delivery, as well as those which had complications post-delivery, because inadequate weight management may have been the cause of this outcome (eFigure 1).

### Measurements

Information for pre-pregnancy height and weight (used for calculating body mass index [BMI] as weight [kg]/height squared [m^2^]) were primarily taken from medical records and completed with self-reports in the mother questionnaire if missing.

Length of gestation was based on treatment date for pregnancies following artificial reproduction technologies and based on last menstrual period date provided by the woman and corrected by early pregnancy ultrasound if discrepancies with the reported last menstrual date were larger than 7 days; for naturally conceived pregnancies, the ultrasound was conducted under 14 weeks of gestation.

Measurements of weight at antenatal visits and their corresponding pregnancy age at measurement were collected at early pregnancy (1 to 15 weeks), mid-pregnancy (16 to 28 weeks), late pregnancy (29 weeks and after), and upon delivery. The early pregnancy visits occurred in average at 11.1 (standard deviation [SD], 1.8) weeks, the mid-pregnancy visits at 23.3 (SD, 2.9) weeks, and the late pregnancy visits before delivery at 32.8 (SD, 3.0) weeks. Weight upon delivery was measured at 38.8 (SD, 1.6) weeks. As the late-pregnancy visit may be close to delivery, some mothers had a missing or same value for either value, in which case only one of the measurements were kept for analysis. Implausible values of weight gain (over 30 kg or under −10 kg) were removed. The outcome, GWG, was calculated as the measurement at each visit/delivery minus pre-pregnancy weight. These represented four values for a woman in 86% of cases. The 14% remaining had one missing value at either the delivery visit (8.0%), the third visit (4.0%), the second visit (1.0%), or the first visit (1.0%).

Pre-pregnancy BMI was divided into categories as recommended by the Institute of Medicine and JSOG: underweight (<18.5 kg/m^2^), normal weight (18.5–24.9 kg/m^2^), overweight (25.0–29.9 kg/m^2^) and obese (≥30 kg/m^2^). Height was defined according to the quartile distribution (≤154, 155–161.9, and ≥162 cm), and maternal age as followed (≤25, 26–34, and ≥35 years). Smoking status was defined based on answers to the questionnaire administered in early pregnancy and categorized as follows: no smoking, quit before pregnancy, quit in early pregnancy, continued smoking. We categorized women into those with and without previous diseases based on reports in the doctor questionnaire at delivery.

### Statistical analysis

#### The Bayesian nonlinear mixed model

Let us note *y_i_*_|_*_j_* the weight gain of the i^th^ woman at the j^th^ week of gestational age. To stabilize variance and ensure a convergence of the model, we specified the model on the logarithmic scale. A constant of 20 was added for all values to ensure positive numbers (prerequisite for using the logarithm scale). The full model follows the form

$$y_{i|j}^{\prime }=\log(y_{i|j}+20)=\beta_{0}+f(GA_{i|j},\beta_{k})+\gamma_{i|j}X_{i|j}^{T}+\tau_{i}+\varepsilon_{i|j}$$

In the Bayesian framework, all parameters are random and require prior distribution.

#### The population-level effects (equivalent to fixed effects in frequentist framework)

*β*_0_ the overall intercept is the expected value of the first women enter in the study. This parameter follows a normal distribution *β*_0_ ∼ *N*(3, 1) with a mean at 3 corresponding to zero weight gain on (exponential scale − 20) with a standard deviation of 1 (2.7 kg). In this study, data were available from the week 1 to the 4^th^ varying according to BMI. Therefore, we specified a realist prior for this parameter to avoid impossible values.

$X_{i|j}^{T}$ is the vector of covariates (maternal characteristics previously listed) with *γ_ij_* ∼ *N*(0, 1).

The function f corresponds to a natural cubic spline depending on gestational age with five degrees of freedom. Other candidates for this function are available but the judgment was based on the background knowledge of the evolution of weight gain during pregnancy, the property of natural cubic splines to extrapolate linearly beyond the boundary knots and the capacity of models to converge. Parameters were modeled with the following prior distributions: *β_k_* ∼ *N*(0, 1).

#### The group-level effects (equivalent to random effects in a frequentist framework)

*τ_i_* represent the deviation in the intercept for the i^th^ woman and follow a cauchy distribution. Between-person residuals $\sigma_{\tau }^{2}$ follow a student distribution. The variance is assumed equal across individuals.

$$\tau_{i}∼N(0,\sigma_{\tau }^{2})\text{ with }\sigma_{\tau }^{2}∼\text{Student\_t}(3,0,1)$$

#### The error term

*ε_i_*_|_*_j_* is the variance of the i^th^ woman at the j^th^ week that is not explained by the model. We assumed a Student distribution for the underweight, normal weight, and overweight population, $\varepsilon_{i|0}∼\text{Student}(0,\sigma_{\varepsilon }^{2})$ with $\sigma_{\varepsilon }^{2}∼\text{Student}(3,0,1)$ , because this distribution tends to be less influenced by outliers.

### Drawing growth curves

For each pre-pregnancy BMI category, we plotted the distribution of GWG by maternal characteristics at four stages of pregnancy (0–15 weeks, 16–27 weeks, 28–37 weeks, and 38–43 weeks). From individual trajectories, in each category, we observed an overall common trend in slope, so we estimated percentile GWG curves from a Bayesian linear mixed-effect model adjusted for maternal age, height, smoking status, previous disease, and a spline function to estimate the effect of gestational age.

The growth curves were drawn by aggregating samples of predicted GWG at each gestational age and smoothing the connecting points. Models were trained and tested on 80% of our population. We kept aside 20% of the data to validate the final curves.

### Sensitivity analysis

Models were re-run with different priors, and we compared different families for the error term. We use the probabilistic programming language Stan implanted in brms package in R version 3.4.0 (R Foundation for Statistical Computing, Vienna, Austria)^8^ to perform this analysis.

### Estimating trajectories required to meet the JSOG guidelines

Based on the growth curve percentages created above, we created ranges of GWG trajectories that would meet the JSOG guidelines that were newly revised in March 2021; that is, 12 to 15 kg for those underweight, 10 to 13 kg for those normal weight, 7 to 10 kg for those overweight, and ≤5 kg for those obese, at 40 weeks of gestation.

As sensitivity analysis, we repeated the analysis to create GWG trajectories required to meet the JSOG guidelines using data on the low-risk population and compared this to those created based on the live-born population.

## RESULTS

### Description of data

Women with a normal weight represented 73.2% of the sample, underweight women 16.1%, overweight 8.2%, and obese women 2.5%. Average weight was 53.1 (SD, 8.8) kg before pregnancy, and women gained on average 10.3 (SD, 4.0) kg at delivery.

In Table 1, we describe GWG at 0–15 weeks, 16–27 week, 28–37 weeks and 38–43 weeks by pre-pregnancy BMI and maternal characteristics. Weight gain was lower for overweight and obese women at all visits compared to normal and underweight women. Younger women, taller women, primiparous women, and women with no previous diseases gained more weight.

**Table 1.** Description of gestational weight gain by maternal characteristics, mean and standard deviation in live-births population

		**Gestational weight gain**			
	Number of woman	0–15 weeks	16–27 weeks	28–37 weeks	38–43 weeks
**Number of data available**		90,396	93,751	104,502	84,126

**Pre-pregnancy BMI <18.5 kg/m^2^**	**15,600**	**0.9 (1.9)**	**5.4 (2.7)**	**8.9 (3.2)**	**11.1 (3.4)**
Maternal age, years					
≤25	2,820	1.2 (2.0)	5.8 (2.8)	9.8 (3.3)	12.3 (3.6)
26 to 34	9,462	0.8 (1.8)	5.3 (2.6)	8.8 (3.1)	11.0 (3.3)
≥35	3,318	0.8 (1.9)	5.2 (2.6)	8.5 (3.1)	10.4 (3.3)
Maternal height, cm					
≤155	3,666	1.0 (1.9)	5.3 (2.6)	8.7 (3.1)	10.9 (3.2)
156 to 161	7,248	0.8 (1.8)	5.4 (2.6)	8.9 (3.2)	11.1 (3.3)
≥162	4,686	0.9 (2.0)	5.5 (2.8)	9.1 (3.4)	11.4 (3.6)
Parity					
Multipara	8,571	0.9 (1.9)	5.4 (2.6)	8.9 (3.1)	11.0 (3.3)
Primipara	7,029	0.9 (1.9)	5.4 (2.7)	9.0 (3.3)	11.3 (3.5)
Smoking status					
Never smoke	8,939	0.5 (1.7)	4.9 (2.4)	8.3 (2.9)	10.5 (3.1)
Quit before realizing pregnancy	3,212	0.9 (1.9)	5.4 (2.6)	9.0 (3.2)	11.2 (3.3)
Quit after realizing pregnancy	2,280	1.7 (2.0)	6.8 (2.8)	10.7 (3.4)	13.1 (3.7)
Smoke during pregnancy	791	2.0 (1.9)	6.6 (2.9)	10.0 (3.5)	12.3 (3.6)
Missing	378	1.1 (1.9)	5.7 (2.7)	9.6 (3.3)	11.8 (3.6)
Previous diseases					
No	11,204	0.9 (1.9)	5.4 (2.6)	9.0 (3.2)	11.2 (3.3)
Yes	4,175	0.9 (1.9)	5.4 (2.7)	8.8 (3.3)	11.0 (3.5)
Missing	221	1.0 (1.9)	5.5 (2.7)	9.4 (3.1)	11.5 (3.5)

**Pre-pregnancy BMI 18.5**–**24.9 kg/m^2^**	**70,684**	**0.6 (2.1)**	**4.9 (2.9)**	**8.5 (3.4)**	**10.8 (3.6)**
Maternal age, years					
≤25	9,191	0.7 (2.2)	5.2 (3.2)	9.2 (3.7)	11.8 (4.0)
26 to 34	41,933	0.5 (2.1)	4.9 (2.9)	8.5 (3.4)	10.8 (3.6)
≥35	19,560	0.6 (2.1)	4.8 (2.9)	8.2 (3.3)	10.2 (3.5)
Maternal height, cm					
≤155	18,278	0.6 (2.0)	4.8 (2.8)	8.3 (3.3)	10.4 (3.5)
156 to 161	34,218	0.5 (2.1)	4.9 (2.9)	8.5 (3.4)	10.8 (3.6)
≥162	18,188	0.6 (2.2)	5.1 (3.1)	8.7 (3.6)	11.1 (3.8)
Parity					
Multipara	41,148	0.5 (2.1)	4.8 (2.9)	8.4 (3.4)	10.5 (3.5)
Primipara	29,536	0.6 (2.1)	5.0 (3.0)	8.6 (3.6)	11.1 (3.8)
Smoking status					
Never smoke	40,467	0.3 (2.0)	4.6 (2.7)	8.0 (3.2)	10.3 (3.4)
Quit before realizing pregnancy	16,746	0.5 (2.1)	4.9 (2.9)	8.5 (3.4)	10.7 (3.5)
Quit after realizing pregnancy	9,033	1.3 (2.3)	6.2 (3.2)	10.1 (3.8)	12.6 (4.1)
Smoke during pregnancy	2,842	1.5 (2.2)	5.8 (3.2)	9.6 (3.9)	11.9 (4.2)
Missing	1,596	0.7 (2.2)	5.2 (3.3)	9.0 (3.7)	11.4 (4.0)
Previous diseases					
No	49,448	0.5 (2.0)	4.9 (2.9)	8.6 (3.4)	10.8 (3.6)
Yes	20,428	0.6 (2.2)	4.8 (3.0)	8.3 (3.6)	10.6 (3.8)
Missing	808	0.6 (2.4)	5.1 (3.2)	9.2 (3.7)	11.3 (4.0)

**Pre-pregnancy BMI 25.0**–**29.9 kg/m^2^**	**7,932**	**0.1 (2.6)**	**3.3 (3.7)**	**6.5 (4.4)**	**8.7 (4.7)**
Maternal age, years					
≤25	967	0.2 (2.9)	3.6 (4.0)	7.4 (4.8)	10.0 (5.1)
26 to 34	4,464	0.1 (2.6)	3.2 (3.7)	6.5 (4.3)	8.6 (4.6)
≥35	2,501	0.3 (2.5)	3.3 (3.5)	6.1 (4.3)	8.1 (4.6)
Maternal height, cm					
≤155	2,292	0.2 (2.5)	3.3 (3.6)	6.5 (4.3)	8.5 (4.5)
156 to 161	3,783	0.1 (2.6)	3.3 (3.6)	6.5 (4.3)	8.7 (4.6)
≥162	1,857	0.2 (2.8)	3.3 (3.9)	6.6 (4.7)	8.8 (5.1)
Parity					
Multipara	5,120	0.1 (2.6)	3.2 (3.5)	6.3 (4.2)	8.3 (4.5)
Primipara	2,812	0.2 (2.7)	3.6 (3.9)	6.8 (4.7)	9.2 (5.0)
Smoking status					
Never smoke	4,019	−0.1 (2.5)	3.0 (3.6)	6.0 (4.3)	8.2 (4.5)
Quit before realizing pregnancy	1,994	0.1 (2.6)	3.2 (3.5)	6.5 (4.1)	8.4 (4.6)
Quit after realizing pregnancy	1,189	0.7 (2.7)	4.2 (3.8)	7.9 (4.7)	10.3 (5.0)
Smoke during pregnancy	492	0.8 (2.9)	3.7 (3.9)	6.9 (4.8)	8.9 (5.2)
Missing	238	0.5 (2.5)	3.7 (3.8)	6.9 (4.4)	9.2 (4.5)
Previous diseases					
No	5,008	0.1 (2.6)	3.4 (3.6)	6.7 (4.3)	8.8 (4.6)
Yes	2,818	0.1 (2.6)	3.1 (3.7)	6.3 (4.5)	8.3 (4.7)
Missing	106	0.4 (2.5)	3.4 (4.0)	7.0 (5.4)	9.6 (5.5)

**Pre-pregnancy BMI ≥30.0 kg/m^2^**	**2,415**	**−0.4 (2.8)**	**1.2 (4.0)**	**3.6 (4.9)**	**5.6 (5.3)**
Maternal age, years					
≤25	267	−0.2 (3.2)	1.5 (4.5)	4.5 (5.2)	6.8 (6.1)
26 to 34	1,374	−0.5 (2.8)	1.1 (3.9)	3.6 (4.7)	5.5 (5.2)
≥35	774	−0.3 (2.7)	1.2 (4.1)	3.3 (4.9)	5.4 (5.1)
Maternal height, cm					
≤155	651	−0.5 (2.7)	1.1 (3.8)	3.6 (4.6)	5.6 (4.8)
156 to 161	1,143	−0.3 (2.8)	1.2 (3.9)	3.5 (4.7)	5.6 (5.2)
≥162	621	−0.3 (2.9)	1.2 (4.4)	3.6 (5.6)	5.8 (5.9)
Parity					
Multipara	1,587	−0.5 (2.7)	1.0 (3.9)	3.4 (4.7)	5.2 (5.0)
Primipara	828	−0.2 (3.0)	1.4 (4.2)	3.9 (5.2)	6.5 (5.7)
Smoking status					
Never smoke	1,082	−0.5 (2.7)	1.0 (3.9)	3.4 (4.6)	5.4 (5.0)
Quit before realizing pregnancy	642	−0.7 (2.9)	0.6 (3.9)	2.9 (4.8)	5.0 (5.1)
Quit after realizing pregnancy	431	0.2 (2.9)	2.3 (4.3)	4.8 (5.2)	7.2 (6.0)
Smoke during pregnancy	178	0.8 (2.7)	2.0 (3.9)	4.1 (4.7)	5.9 (5.2)
Missing	82	−0.6 (3.1)	0.6 (4.1)	3.5 (5.3)	5.1 (5.5)
Previous diseases					
No	1,372	−0.3 (2.8)	1.3 (4.0)	3.7 (4.7)	5.9 (5.3)
Yes	1,006	−0.4 (2.7)	1.0 (4.0)	3.3 (5.0)	5.4 (5.3)
Missing	37	−0.9 (3.8)	0.9 (4.4)	4.4 (6.2)	4.8 (6.2)

BMI, body mass index.

### The mixed model in Bayesian framework

The full posterior distribution was estimated with Markov chain Monte Carlo (MCMC) simulations; simulated draws for each observations were made from four chains, with the goal of 52,000 samples for underweight and 60,000 samples for obesity and overweight groups to reach a minimum of 10,000 for the effective sample size (ESS), the number recommended for an accurate highest density interval (HDI).^13^ We successfully reached this condition with the normal weight group, but we faced a memory issue to compute predictive posteriors, with the computer lacking capacity to fully perform the model on the whole sample. Hence, we reduced the sample size to 12,000, which impacts ESS but not estimators and HDI. Convergence, posterior predictive checks (eFigure 2), residual plots (eFigure 3), and Quantile-Quantile plots (eFigure 4) were examined. GWG trajectories in the validation dataset fit well with the estimated curves (eFigure 5).

Parameters were summarized in term of median and HDI in Table 2. As we log-transform the dependent variable, this implies that independent variables have a multiplicative relationship with the dependent variable, as can be seen when we exponentiate our initial model. Under the ceteris paribus assumption, underweight and normal weight younger (under age 25) women had a weight gain 1.023 times and 1.015 times higher than older (age 35 and over) women, but 0.987 and 0.980 times lower in overweight and obese populations. Shorter women (155 cm and under) had a weight gain 0.991 and 0.986 times lower than taller women in underweight and normal weight groups.

**Table 2.** Main effects of Bayesian mixed models (splines excluded) in live-births population

	Estimate median	Exponential of Estimated median	Estimated Error	95% HDI	ESS
**Number of post-warmup samples (52,000)**		**Pre-pregnancy BMI <18.5 kg/m^2^**			

Maternal age, years					
≤25	0.023	1.023	0.003	[0.018, 0.028]	16,600
26 to 34	0.008	1.008	0.002	[0.004, 0.012]	15,355
≥35	ref.				
Maternal height, cm					
≤155	−0.009	0.991	0.002	[−0.013, −0.005]	16,741
156 to 161	−0.007	0.993	0.002	[−0.010, −0.003]	16,165
≥162	ref.				
Parity					
Multipara	ref.				
Primipara	−0.001	0.999	0.002	[−0.004, 0.002]	16,262
Smoking status					
Never smoke	ref.				
Quit before realizing pregnancy	0.020	1.020	0.002	[0.016, 0.023]	15,647
Quit after realizing pregnancy	0.070	1.073	0.002	[0.066, 0.075]	16,324
Smoke during pregnancy	0.060	1.062	0.004	[0.054, 0.067]	16,696
Previous diseases					
No	ref.				
Yes	−0.003	0.997	0.002	[−0.006, 0.001]	17,192
sd(Intercept)	0.080		0.001	[0.078, 0.081]	22,310

**Number of post-warmup samples (12,000)**		**Pre-pregnancy BMI 18.5–24.9 kg/m^2^**			

Maternal age, years					
≤25	0.015	1.015	0.001	[0.012, 0.018]	5,691
26 to 34	0.004	1.004	0.001	[0.002, 0.006]	6,267
≥35	ref.				
Maternal height, cm					
≤155	−0.014	0.986	0.001	[−0.016, −0.012]	5,972
156 to 161	−0.006	0.994	0.001	[−0.008, −0.004]	6,203
≥162	ref.				
Parity					
Multipara	ref.				
Primipara	0.006	1.006	0.001	[0.004, 0.007]	6,822
Smoking status					
Never smoke	ref.				
Quit before realizing pregnancy	0.014	1.014	0.001	[0.012, 0.016]	5,886
Quit after realizing pregnancy	0.063	1.065	0.001	[0.060, 0.065]	6,070
Smoke during pregnancy	0.050	1.051	0.002	[0.046, 0.054]	5,948
Previous diseases					
No	ref.				
Yes	−0.005	0.995	0.001	[−0.007, −0.004]	6,096
sd(Intercept)	0.094		0.000	[0.093, 0.094]	7,472

**Number of post-warmup samples (60,000)**		**Pre-pregnancy BMI 25.0–29.9 kg/m^2^**			

Maternal age, years					
≤25	−0.013	0.987	0.006	[−0.024, −0.001]	14,206
26 to 34	−0.015	0.985	0.006	[−0.027, −0.003]	14,730
≥35	ref.				
Maternal height, cm					
≤155	0.003	1.003	0.004	[−0.006, 0.011]	13,474
156 to 161	0.002	1.002	0.005	[−0.008, 0.012]	14,028
≥162	ref.				
Parity					
Multipara	ref.				
Primipara	0.014	1.014	0.004	[0.007, 0.022]	14,541
Smoking status					
Never smoke	ref.				
Quit before realizing pregnancy	0.013	1.013	0.004	[0.004, 0.021]	15,314
Quit after realizing pregnancy	0.053	1.054	0.005	[0.042, 0.063]	13,883
Smoke during pregnancy	0.025	1.025	0.008	[0.01, 0.04]	14,891
Previous diseases					
No	ref.				
Yes	−0.014	0.986	0.004	[−0.021, −0.006]	13,889
sd(Intercept)	0.137		0.001	[0.135, 0.140]	21,714

**Number of post-warmup samples (60,000)**		**Pre-pregnancy BMI ≥30.0 kg/m^2^**			

Maternal age, years					
≤25	−0.020	0.980	0.014	[−0.047, 0.007]	15,617
26 to 34	−0.015	0.985	0.015	[−0.044, 0.014]	15,261
≥35	ref.				
Maternal height, cm					
≤155	−0.002	0.998	0.010	[−0.021, 0.018]	15,241
156 to 161	−0.010	0.990	0.011	[−0.032, 0.013]	15,696
≥162	ref.				
Parity					
Multipara	ref.				
Primipara	0.024	1.024	0.009	[0.006, 0.042]	14,211
Smoking status					
Never smoke	ref.				
Quit before realizing pregnancy	−0.030	0.970	0.01	[−0.050, −0.011]	15,733
Quit after realizing pregnancy	0.042	1.043	0.012	[0.019, 0.064]	15,576
Smoke during pregnancy	0.034	1.035	0.017	[0.001, 0.067]	16,429
Previous diseases					
No	ref.				
Yes	−0.013	0.987	0.009	[−0.03, 0.004]	15,303
sd(Intercept)	0.174		0.003	[0.168, 0.180]	16,658

BMI, body mass index; ESS, effective sample size; HDI, highest probability density (indicates which points of a distribution are most credible. 10,000 independent steps are recommended for an accurate 95% HDI).

Outcome of model is log-transformed weight gain (kg).

Primipara women gained more weight than multipara women, with a larger difference among women with higher pre-pregnancy BMI. Women that quit smoking after realizing pregnancy had a weight gain 1.043 to 1.073 times higher than non-smoking women, while the difference with those continuing to smoke during pregnancy were slightly lower (1.025 to 1.062).

### Growth curves

**Figure 1.**
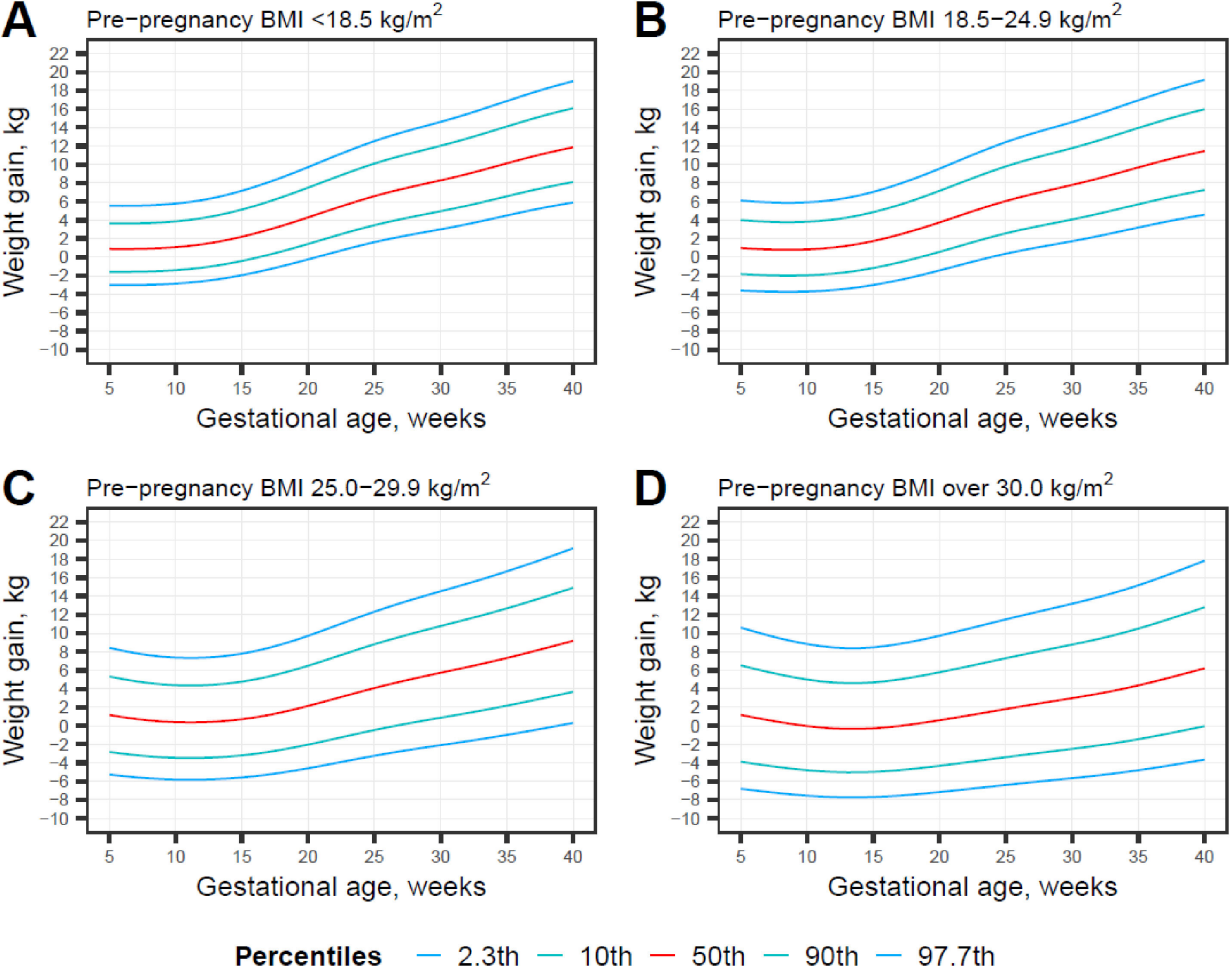
Gestational weight gain percentile charts for underweight (A), normal weight (B), overweight (C) and obese (D) populations.

In Figure 1, we show GWG percentiles drawn from predictive distribution for underweight, normal weight, overweight, and obese women among the whole sample based on the Bayesian model. Corresponding percentile values for each week of gestation are shown in eTable 1. GWG curves were substantially different by pre-pregnancy BMI category. The estimated 50^th^ percentiles at 10 weeks, 20 weeks, 30 weeks, and 40 weeks for underweight women were 1.1 (10^th^–09^th^ percentile, −1.4 to 3.9) kg, 4.3 (10^th^–09^th^ percentile, 1.4–7.5) kg, 8.3 (10^th^–09^th^ percentile, 5.0–12.0) kg, and 11.9 (10^th^–09^th^ percentile, 8.1–16.1) kg. Similarly, for normal-weight women, the 50^th^ percentile values were 0.8 (10^th^–09^th^ percentile, −2.0 to 3.8) kg, 3.8 (10^th^–09^th^ percentile, 0.6–7.2) kg, 7.8 (10^th^–09^th^ percentile, 4.1–11.8) kg and 11.5 (10^th^–09^th^ percentile, 7.2–16.0) kg; for overweight women, the values were 0.4 (10^th^–09^th^ percentile, −3.5 to 4.4) kg, 2.1 (10^th^–09^th^ percentile, −2.0 to 6.5) kg, 5.7 (10^th^–09^th^ percentile, 0.9–10.8) kg, and 9.2 (10^th^–09^th^ percentile, 3.7–14.9) kg; and for obese women, the values were 0.0 (10^th^–09^th^ percentile, −4.8 to 5.0) kg, 0.6 (10^th^–09^th^ percentile, −4.3 to 5.8) kg, 3.0 (10^th^–09^th^ percentile, −2.5 to 8.8) kg, and 6.2 (10^th^–09^th^ percentile, 0.0–12.8) kg.

**Figure 2.**
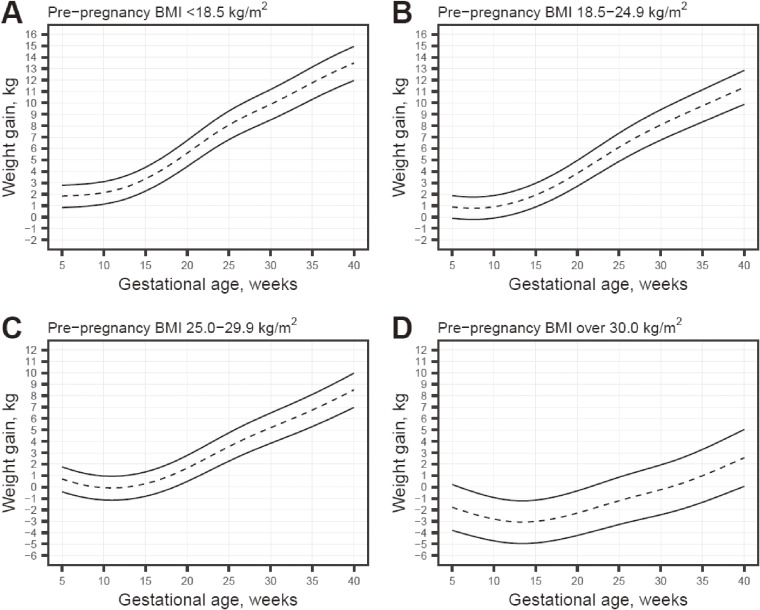
Range of gestational weight gain of trajectories that will meet the JSOG recommendation (2021) at 40 weeks of gestation for underweight (A), normal weight (B), overweight (C) and obese (D) populations. JSOG, Japan Society of Obstetrics and Gynecology

In Figure 2, we show the trajectory curves that lead to the JSOG recommendation of GWG at 40 weeks of gestation based these GWG percentiles. Corresponding ranges for each week of gestation are shown in eTable 2. Among underweight women, the estimated range of GWG that led to 12 to 15 kg at 40 weeks was 1.2 to 3.2 kg at 10 weeks, 4.4 to 6.7 kg at 20 weeks, and 8.4 to 11.1 kg at 30 weeks. Similarly, for normal-weight women, the ranges that led to 10 to 13 kg at 40 weeks was −0.2 to 1.8 kg at 10 weeks, 2.6 to 4.9 kg at 20 weeks, and 6.4 to 9.1 kg at 30 weeks. For overweight women, the ranges that led to 7 to 10 kg at 40 weeks were −1.2 to 0.9 kg at 10 weeks, 0.4 to 2.7 kg at 20 weeks, and 3.8 to 6.5 kg at 30 weeks; and for obese women, the range that led to under 5 kg of GWG at 40 weeks was <−0.9 kg at 10 weeks, <−0.3 kg at 20 weeks and <1.9 kg at 30 weeks.

Trajectory curves based on the low risk population were similar to those based on the whole population are shown in eFigure 6 (except for at under 10 weeks for the obese population, for which the estimated weight gain was lower in the low-risk population). Description of GWG by maternal characteristics, as well as main effects of maternal characteristics for this low-risk population, are shown in eTable 3 and eTable 4. Ranges of GWG for each week of gestation corresponding to eFigure 6 are shown in eTable 5.

## DISCUSSION

The purpose of this study was to produce GWG growth charts for the Japanese population by pre-pregnancy BMI, as well as show the range of GWG that lead to the JSOG recommendation of GWG at delivery. GWG percentile curves were substantially different by pre-pregnancy BMI category. Our GWG curves estimated that underweight, normal weight, overweight, and obese women who gain 8.5 to 11.2 kg, 6.7 to 9.4 kg, 3.8 to 6.5 kg, and <2.3 kg at 30 weeks of gestation are on the trajectory to reach GWG as recommended in the new guidelines at 40 weeks of gestation.

Few previous studies on GWG reference charts exist in the Asian population. A recent literature review identified two studies,^14^^,^^15^ both conducted 20 years ago. The Ochsenbein-Kolble’s study was a cross-sectional study based on data from 1996 to 2000 that included Asian women delivering in Zurich University Hospital (Switzerland). Women were mostly born in Sri Lanka, Thailand, and the Philippines (*N* = 578), with a mean weight slightly higher than our sample (55.3 kg; SD, 10.1 kg vs 52.0; SD, 7.3 kg). They did not present data stratified by BMI; however, we can see the weight gain curve of the Asian (and Black) population is lower than that of the Caucasians population, with a difference of 2 kg. Wong’s study was conducted in 1997 on the Chinese population (*N* = 504) in Hong Kong. The method used was unclear, but they found that the target weight gain should be 13 to 16.7 kg, 11 to 16.4 kg, and 7.1 to 14.4 kg for underweight, normal weight, and overweight, respectively. A visual inspection (no raw data available) shows that Chinese women seems to have a 50th percentile slightly higher after 4 months of pregnancy, but it seems to correspond to other studies at the end of delivery. These studies suggest that Asians in general have a lower GWG compared to Caucasians and suggest that more research in this population is needed.

The largest study on GWG curves up to date was conducted by Santos et al.^7^ They used longitudinal data of low-risk Caucasian women from multiple cohorts in Europe, the United States, and Oceania. A second recent study, based on the Intergrowth 21^th^ project, produced GWG growth charts for normal-weight women residing in urban regions in Brazil, China, India, Italy, Kenya, Oman, the United Kingdom, and the United States. Upon comparing the median GWG for the normal population, we observed a significantly lower weight gain (10.9; interquartile range, 8.7–13.2) at 40 weeks of gestation in our study compared to 13.7 kg (interquartile range, 10.9–16.9 kg) reported in the Intergrowth 21^th^ project, and 14.5 kg (interquartile range, 11.5–17.7 kg) reported in Santos’s study, providing additional evidence for the merit of creating GWG curves specifically for Japanese.

In this study, we directed our analysis towards the Bayesian framework instead of the GAMLSS method, the most common method to produce reference curves. The GAMLSS method assumes that all observations are independent, and it corrects the non-normality of the outcome by applying a correction based on multiple parameters depending on the skewness and kurtosis of the outcome, in addition to location and scale. Compared to the GAMLSS method, the Bayesian framework has a large choice of distribution families, and it is able to explore complex models to examine possible influence of certain explanatory variables on the outcome. In addition, it produces several samples from which the percentiles can be estimated. Integration of prior information in models, especially for small datasets (as was the case for the overweight and obese populations) and unbalanced designs, have helped to improve convergence and accuracy of models.^16^

While we created GWG percentiles only stratified by pre-pregnancy BMI, we also investigated how different maternal characteristics influenced GWG. We observed that weight gain differs largely by smoking status, and less by maternal age and height. Women who smoked during early pregnancy and beyond consistently had higher GWG compared to non-smokers and those who quit smoking before pregnancy.

On the other hand, the influence of maternal height and age was limited to 1–2% of GWG, suggesting that the additional value of creating customized growth charts based on these characteristics may be limited.

### GWG trajectories in relation to GWG recommendations

Based on the GWG percentile curves created in our analysis, we estimated the percentiles that led to the recommended range of GWG at delivery. We could not identify any previous studies that had calculated and proposed such curves. While the utility of such curves is to be determined by future research, as previous research have repetitively shown how difficult it is to have women gain the recommended range (only 35% of the live-birth population had GWG trajectories within the range of the JSOG guidelines at 40 weeks, with the percentage ranging from 29% among women underweight to 43% among those overweight and obese), we think our results and similar curves may help pregnant women track their weight during pregnancy, making it possible to optimize pregnancy outcomes.

### Strengths and limitations of the study

This study is based on nationwide birth cohort, so data are well representative of the Japanese population, suggesting our GWG percentiles curves are likely to be generalizable to the general population. Based on trajectories of GWG in the low-risk population, we also were able to estimate the range of GWG for each week of gestation that are on the trajectory that leads to meeting the JSOG recommendation at delivery, which may be help pregnant women monitor weight during their pregnancy.

In our study, we applied Bayesian modeling to create GWG weight gain percentiles curves for Japanese women. Bayesian modeling has great flexibility, regardless of the distribution, and can accommodate multiple distributional families, taking into account dependence of observations, multiple factors, and covariance structure. While checking each model requires time and consumes a lot of data memory, Bayesian analysis is now available in many software programs. We think this method should be considered more often, especially when researchers are interested in inclusion of covariates.

However, our study has several limitations. First, we calculated GWG based on self-reported pre-pregnancy weight, so misreporting would lead to miscalculation of GWG. Social desirability bias would have caused overestimation of GWG if more women reported their pre-pregnancy weight to be lower than what is true. Second, data on GWG was not collected in the same conditions during each pregnancy check-up. Ideally, the weight should be collected in the same time for all women or at least in a reasonable range; however, in this study, some women only had sparse measurements, with long intervals between two visits making it difficult to understand the real progress in weight gain. While we tried to overcome this handicap using statistical modeling, as Bayesian mixed models can deal with this issue, it would be ideal to have more equally distributed measurements. Third, while we calculated the range of GWG trajectories that lead to meeting the revised GWG recommendations, this depends on the assumption that women’s characteristics other than GWG have not changed since 2011–2014. Thus, future studies on women undergoing pregnancy under the revised GWG recommendations are required to check the validity of our findings.

## SUPPLEMENTARY MATERIAL

eFigure 1. Flow chart

eFigure 2. Distributions based on training dataset of logarithm gestational weight gain observed (y) and estimated (yrep) using a sample of 10 posterior distributions; models adjusted for maternal characteristics for underweight (A), normal weight (B), overweight (C) and obese (D) populations. Underweight, normal weight, overweight, and obese population followed a Student distribution.

eFigure 3. Residuals versus estimated logarithm gestational weight gain based on the training datasets; models adjusted for maternal characteristics for underweight (A), normal weight (B), overweight (C) and obese (D) populations. Underweight, normal weight, overweight, and obese populations followed a Student distribution.

eFigure 4. Q-Q plot of Studentized residuals based on the training datasets, models adjusted for maternal characteristics for underweight (A), normal weight (B), overweight (C) and obese (D) populations. Underweight, normal weight, overweight, and obese population followed a Student distribution.

eFigure 5. Individual trajectories of gestational weight gain of the validation set overlaid with estimated gestational weight gain percentiles curves for underweight (A), normal weight (B), overweight (C) and obese (D) populations.

eFigure 6. Range of gestational weight gain of trajectories that will meet the JSOG recommendation (2021) at 40 weeks of gestation for underweight (A), normal weight (B), overweight (C) and obese (D) populations based on GWG curves calculated from the live-born population (dashed line) and the low-risk population (solid line)

eTable 1. Percentiles of gestational weight gain in kilograms among live births

eTable 2. Range of gestational weight gain in kilograms to meet current guidelines (based on GWG curves based on the live-born population)

eTable 3. Description of gestational weight gain by maternal characteristics; mean and standard deviation in low-risk population

eTable 4. Main effects of Bayesian mixed models (splines excluded) in low-risk population

eTable 5. Range of gestational weight gain in kilograms to meet current guidelines (based on GWG curves based on the low risk population)
