# Supplementary material for: Gestational Weight Gain Growth Charts Adapted to Japanese Pregnancies Using a Bayesian Approach in a Longitudinal Study: The Japan Environment and Children’s Study
Source: J Epidemiol. 2023 May 5;33(5):217–26. doi: 10.2188/jea.JE20210049 (PMC10043156; doi:10.2188/jea.JE20210049)
Supplement: Supplementary file 1 [file je-33-217-s001.pdf]

103,099 Pregnancies

2,349 Those with unknown gestational age at term

1,544 Stillbirth or unknown outcome

2,575 Those with less than 3 weight measures or measurement errors

96,631 Live birth with valid data

41,423 Not a spontaneous vaginal delivery

74 Multiple births and births missing record on multiplicity

1,865 Preterm pregnancies

24,735 Either pregnancy or obstetric complications

5,281 Problem of placenta

1,792 Congenital disease of child

1,830 Problem with baby at 1 month

1,681 Problem with mother at 1 month

17,950 Women with a low risk outcome for the child and mother

eFigure 1. Flow chart

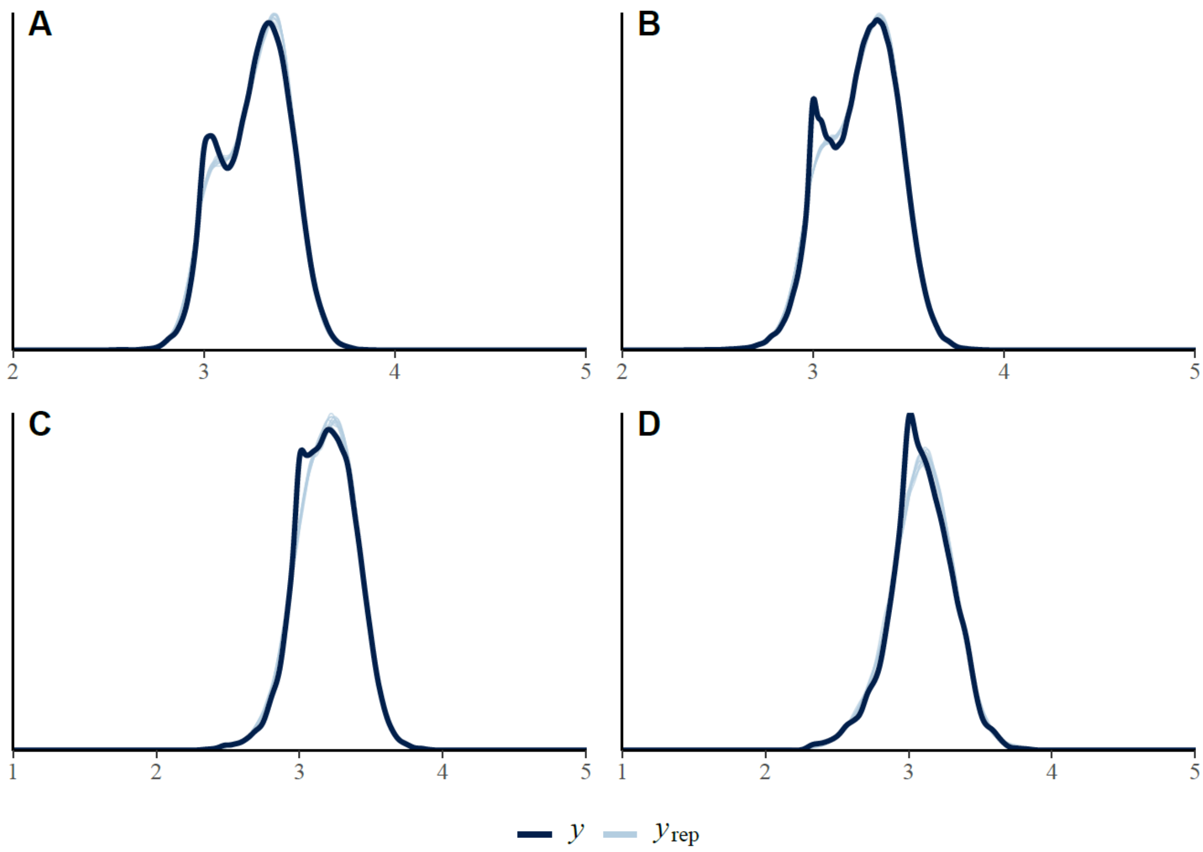

eFigure 2. Distributions based on training dataset of logarithm gestational weight gain observed (y) and estimated (yrep) using a sample of 10 posterior distributions; models adjusted for maternal characteristics for underweight (A), normal weight (B), overweight (C) and obese (D) populations. Underweight, normal weight, overweight, and obese population followed a Student distribution.

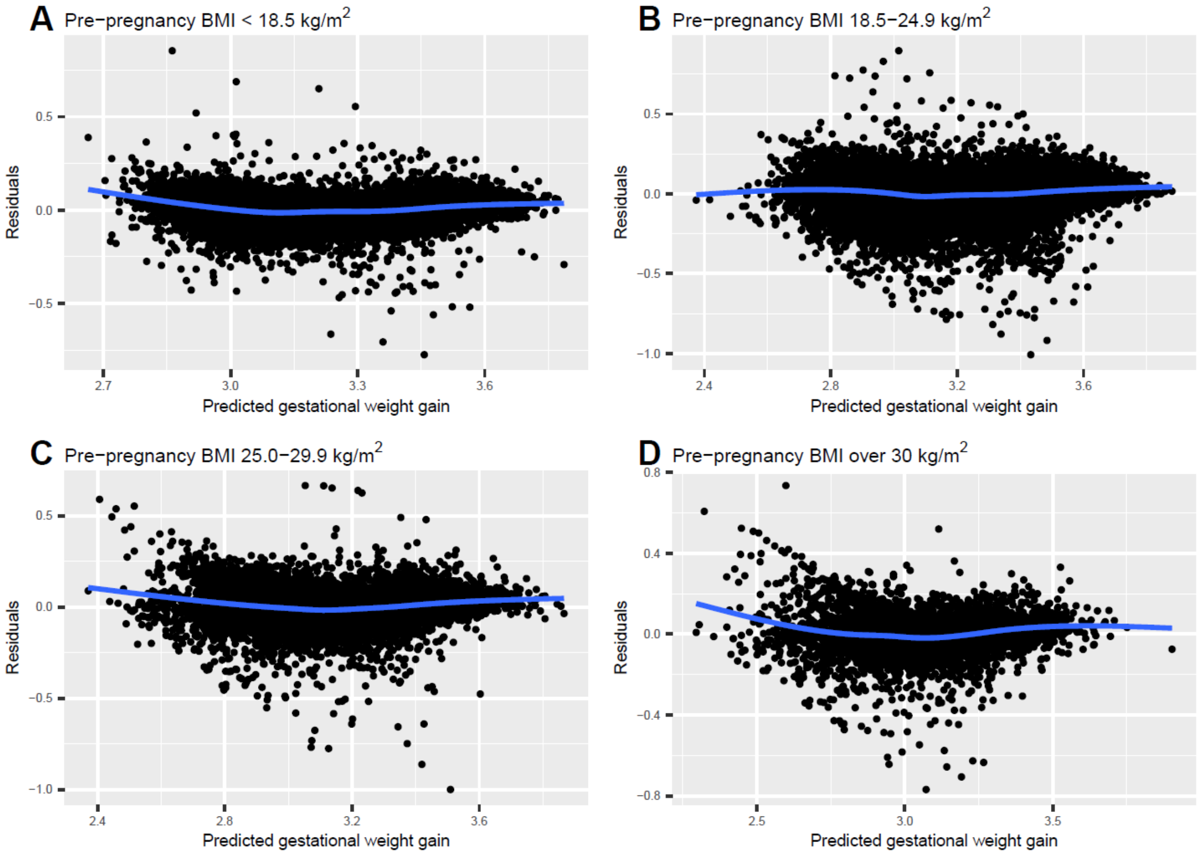

eFigure 3. Residuals versus estimated logarithm gestational weight gain based on the training datasets; models adjusted for maternal characteristics for underweight (A), normal weight (B), overweight (C) and obese (D) populations. Underweight, normal weight, overweight, and obese populations followed a Student distribution.

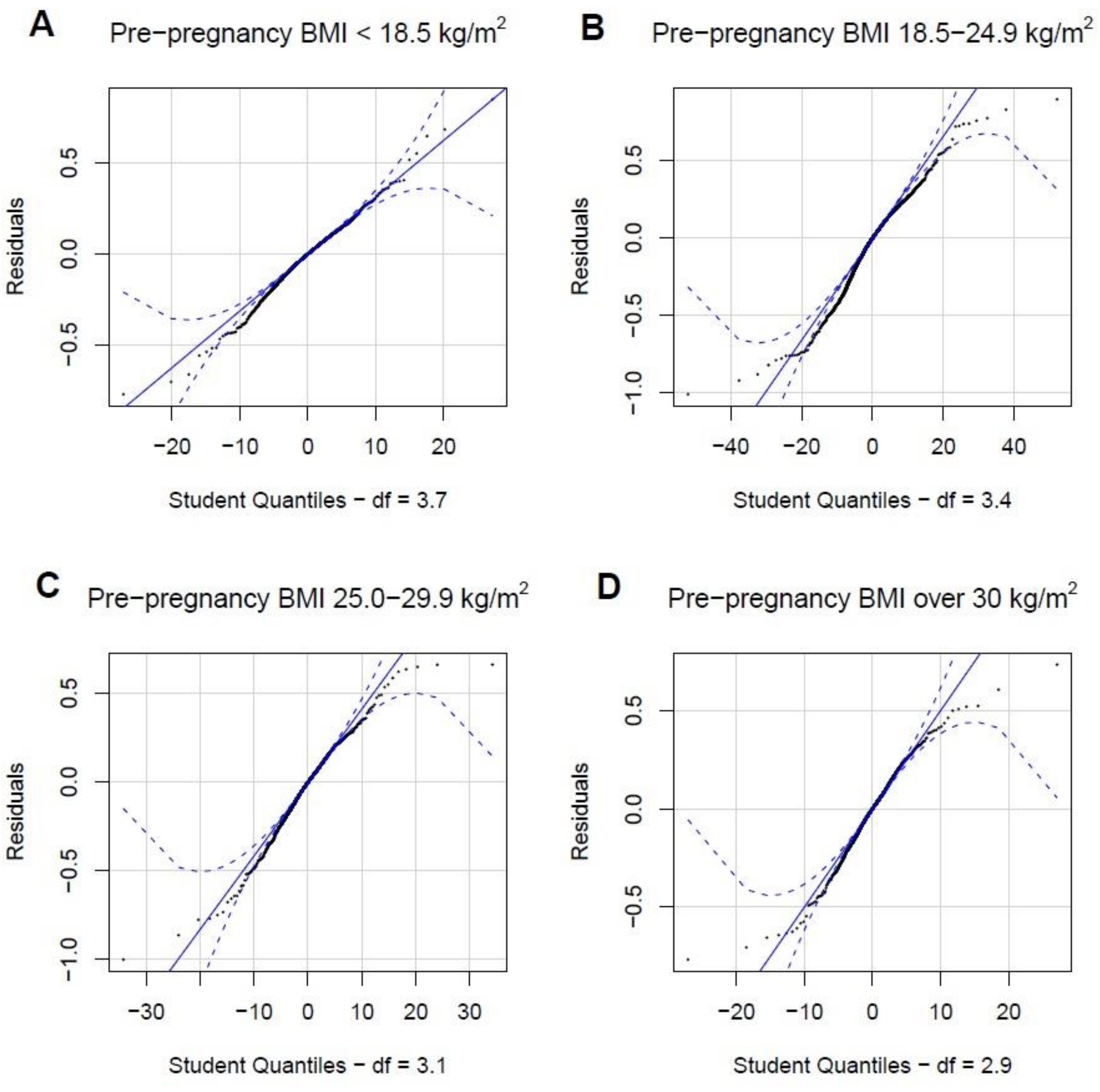

eFigure 4. Q-Q plot of Studentized residuals based on the training datasets, models adjusted for maternal characteristics for underweight (A), normal weight (B), overweight (C) and obese (D) populations. Underweight, normal weight, overweight, and obese population followed a Student distribution.

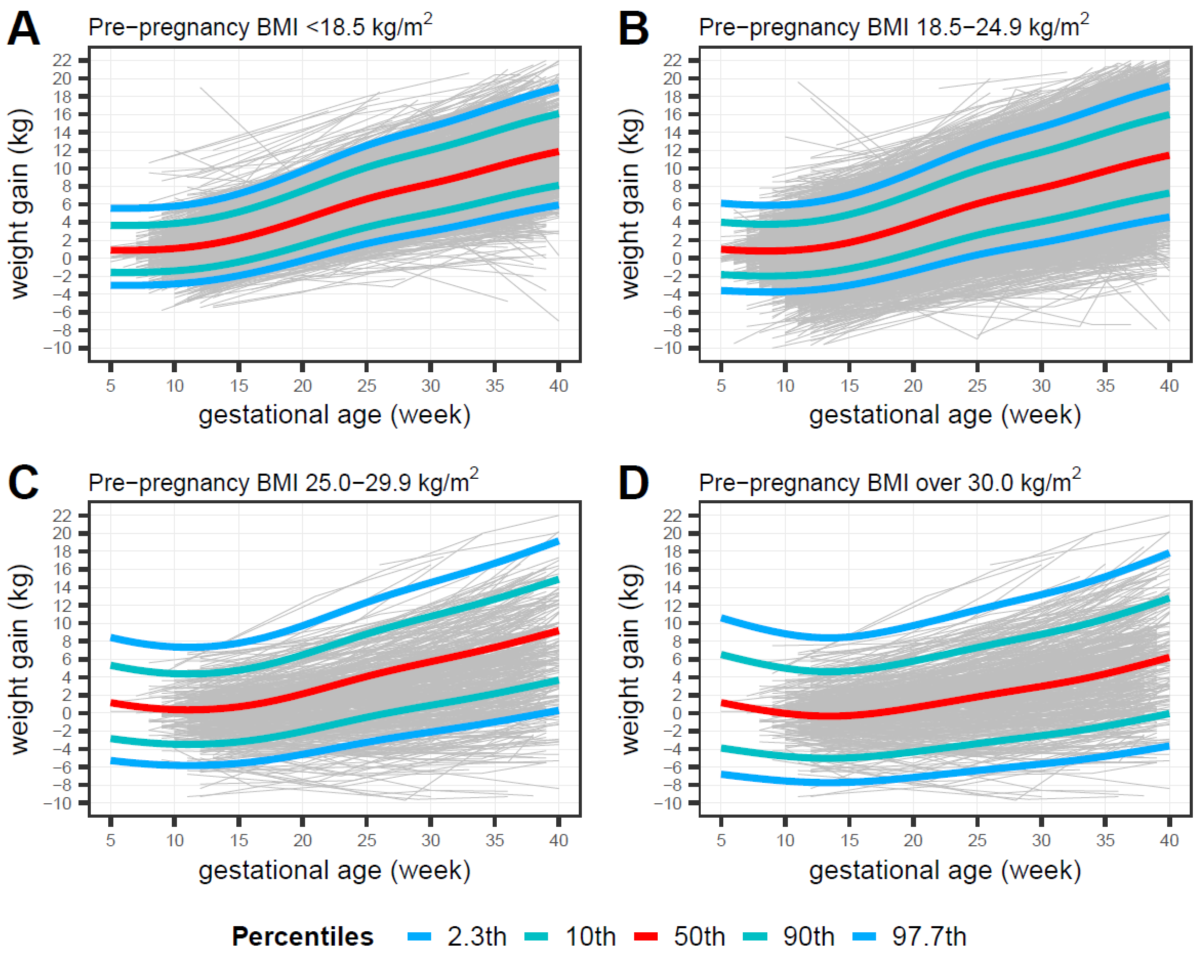

eFigure 5:. Individual trajectories of gestational weight gain of the validation set overlaid with estimated gestational weight gain percentiles curves for underweight (A), normal weight (B), overweight (C) and obese (D) populations.

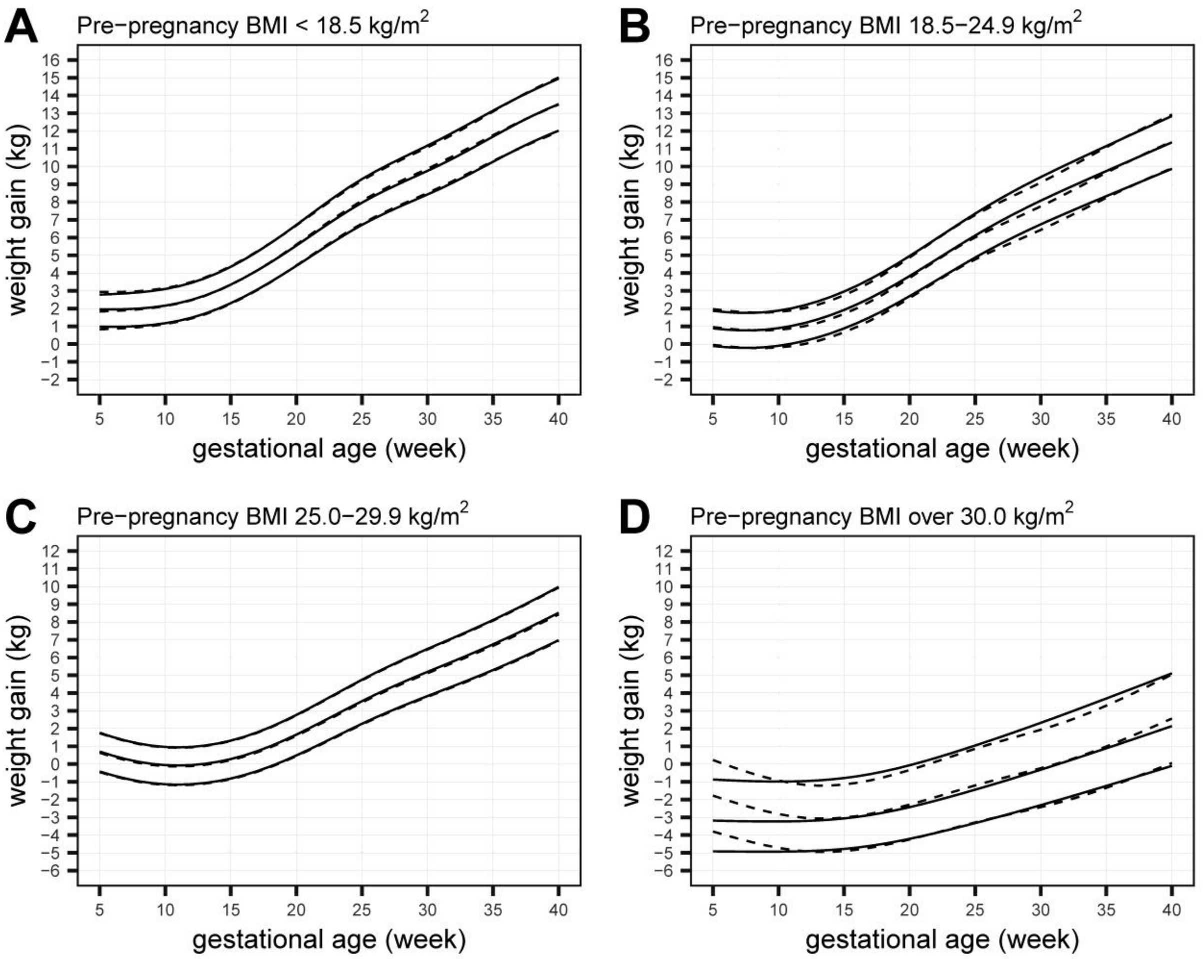

eFigure 6. Range of gestational weight gain of trajectories that will meet the JSOG recommendation (2021) at 40 weeks of gestation for underweight (A), normal weight (B), overweight (C) and obese (D) populations based on GWG curves calculated from the live-born population (dashed line) and the low-risk population (solid line)

eTable 1. Percentiles of gestational weight gain in kilograms among live births

A. Underweight women

Gestational week	1%	2.3%	10%	25%	50%	75%	90%	97.7%	99%
5	-3.764	-3.024	-1.581	-0.418	0.885	2.268	3.648	5.554	6.621
6	-3.771	-3.031	-1.588	-0.426	0.877	2.259	3.638	5.543	6.610
7	-3.763	-3.022	-1.579	-0.416	0.887	2.269	3.649	5.555	6.622
8	-3.736	-2.995	-1.550	-0.385	0.920	2.304	3.686	5.595	6.662
9	-3.688	-2.945	-1.496	-0.327	0.981	2.369	3.755	5.670	6.738
10	-3.614	-2.868	-1.413	-0.238	1.076	2.469	3.862	5.786	6.856
11	-3.510	-2.759	-1.296	-0.114	1.208	2.610	4.011	5.948	7.024
12	-3.373	-2.615	-1.140	0.051	1.384	2.798	4.211	6.163	7.248
13	-3.197	-2.432	-0.942	0.262	1.609	3.037	4.465	6.438	7.535
14	-2.984	-2.209	-0.701	0.518	1.882	3.330	4.775	6.773	7.885
15	-2.735	-1.949	-0.420	0.817	2.201	3.669	5.135	7.162	8.293
16	-2.456	-1.657	-0.104	1.153	2.559	4.052	5.541	7.601	8.752
17	-2.150	-1.337	0.243	1.521	2.952	4.472	5.987	8.082	9.256
18	-1.820	-0.993	0.617	1.919	3.376	4.924	6.467	8.600	9.798
19	-1.470	-0.628	1.013	2.340	3.825	5.403	6.975	9.150	10.372
20	-1.105	-0.247	1.427	2.779	4.294	5.902	7.506	9.724	10.970
21	-0.733	0.141	1.849	3.228	4.773	6.412	8.048	10.310	11.581
22	-0.360	0.530	2.271	3.677	5.252	6.923	8.590	10.896	12.190
23	0.005	0.911	2.685	4.117	5.721	7.423	9.121	11.470	12.787
24	0.355	1.276	3.082	4.539	6.171	7.902	9.630	12.019	13.359
25	0.681	1.617	3.452	4.933	6.591	8.349	10.105	12.533	13.893
26	0.979	1.928	3.790	5.292	6.973	8.757	10.538	13.000	14.380
27	1.251	2.214	4.099	5.621	7.324	9.131	10.935	13.429	14.827
28	1.508	2.483	4.390	5.930	7.654	9.483	11.308	13.832	15.246
29	1.756	2.743	4.672	6.230	7.973	9.824	11.670	14.222	15.653
30	2.004	3.004	4.955	6.530	8.293	10.165	12.033	14.613	16.061
31	2.261	3.274	5.247	6.840	8.624	10.519	12.408	15.018	16.483
32	2.535	3.560	5.558	7.171	8.977	10.895	12.807	15.449	16.932
33	2.825	3.864	5.888	7.522	9.351	11.294	13.231	15.906	17.408
34	3.124	4.178	6.229	7.884	9.738	11.706	13.669	16.380	17.901
35	3.427	4.495	6.574	8.251	10.129	12.123	14.112	16.859	18.400
36	3.727	4.808	6.914	8.614	10.516	12.535	14.550	17.333	18.893

37	4.016	5.111	7.243	8.964	10.889	12.933	14.972	17.790	19.370
38	4.288	5.396	7.553	9.293	11.241	13.307	15.370	18.221	19.819
39	4.541	5.660	7.839	9.598	11.566	13.654	15.739	18.621	20.236
40	4.776	5.907	8.106	9.882	11.869	13.977	16.082	18.993	20.625

B. Normal weight women

Gestational week	1%	2.3%	10%	25%	50%	75%	90%	97.7%	99%
5	-4.505	-3.601	-1.824	-0.470	0.988	2.494	3.999	6.116	7.330
6	-4.570	-3.670	-1.900	-0.552	0.900	2.400	3.898	6.006	7.216
7	-4.617	-3.720	-1.956	-0.611	0.836	2.332	3.824	5.926	7.131
8	-4.643	-3.747	-1.987	-0.644	0.800	2.294	3.784	5.881	7.083
9	-4.645	-3.749	-1.989	-0.646	0.798	2.292	3.781	5.878	7.078
10	-4.618	-3.720	-1.958	-0.612	0.835	2.331	3.823	5.922	7.123
11	-4.560	-3.658	-1.890	-0.539	0.914	2.416	3.913	6.020	7.224
12	-4.467	-3.559	-1.781	-0.421	1.040	2.552	4.057	6.176	7.387
13	-4.336	-3.419	-1.626	-0.255	1.218	2.743	4.261	6.398	7.619
14	-4.163	-3.237	-1.424	-0.038	1.452	2.993	4.529	6.689	7.925
15	-3.952	-3.014	-1.176	0.227	1.737	3.299	4.855	7.045	8.299
16	-3.706	-2.755	-0.889	0.535	2.069	3.654	5.235	7.459	8.734
17	-3.430	-2.464	-0.566	0.882	2.441	4.053	5.661	7.924	9.223
18	-3.128	-2.144	-0.212	1.262	2.850	4.491	6.129	8.433	9.759
19	-2.803	-1.800	0.169	1.671	3.290	4.962	6.631	8.980	10.334
20	-2.460	-1.437	0.571	2.103	3.754	5.459	7.162	9.557	10.940
21	-2.106	-1.062	0.985	2.548	4.232	5.972	7.709	10.152	11.563
22	-1.751	-0.685	1.402	2.996	4.714	6.488	8.259	10.750	12.190
23	-1.402	-0.314	1.812	3.437	5.187	6.995	8.799	11.337	12.805
24	-1.067	0.041	2.204	3.859	5.640	7.481	9.318	11.901	13.394
25	-0.756	0.371	2.570	4.253	6.063	7.934	9.801	12.426	13.942
26	-0.473	0.670	2.903	4.610	6.448	8.346	10.240	12.905	14.441
27	-0.213	0.945	3.209	4.939	6.802	8.725	10.645	13.345	14.900
28	0.033	1.205	3.498	5.250	7.136	9.083	11.027	13.762	15.334
29	0.273	1.458	3.781	5.554	7.463	9.433	11.401	14.170	15.758
30	0.516	1.715	4.067	5.862	7.793	9.787	11.779	14.582	16.188
31	0.770	1.985	4.366	6.183	8.139	10.157	12.175	15.013	16.639
32	1.043	2.273	4.686	6.526	8.507	10.552	12.597	15.472	17.121

33	1.329	2.576	5.021	6.886	8.894	10.967	13.040	15.954	17.628
34	1.623	2.887	5.365	7.255	9.291	11.393	13.494	16.447	18.147
35	1.918	3.199	5.709	7.626	9.689	11.819	13.950	16.943	18.668
36	2.208	3.505	6.048	7.990	10.079	12.238	14.397	17.429	19.179
37	2.486	3.799	6.374	8.339	10.455	12.641	14.827	17.897	19.669
38	2.748	4.076	6.680	8.668	10.808	13.020	15.231	18.337	20.128
39	2.995	4.336	6.970	8.979	11.142	13.377	15.613	18.753	20.560
40	3.230	4.583	7.245	9.275	11.459	13.717	15.976	19.149	20.970

C. Overweight women

Gestational week	1%	2.3%	10%	25%	50%	75%	90%	97.7%	99%
5	-6.436	-5.278	-2.833	-0.880	1.162	3.238	5.318	8.415	10.285
6	-6.584	-5.437	-3.020	-1.088	0.931	2.984	5.040	8.102	9.950
7	-6.710	-5.574	-3.180	-1.267	0.732	2.766	4.801	7.833	9.662
8	-6.813	-5.685	-3.310	-1.412	0.572	2.589	4.608	7.614	9.429
9	-6.888	-5.766	-3.406	-1.518	0.454	2.459	4.466	7.454	9.258
10	-6.934	-5.816	-3.465	-1.584	0.381	2.379	4.378	7.355	9.152
11	-6.951	-5.835	-3.487	-1.609	0.354	2.349	4.345	7.318	9.112
12	-6.939	-5.821	-3.472	-1.592	0.372	2.369	4.367	7.342	9.138
13	-6.897	-5.776	-3.420	-1.534	0.436	2.440	4.444	7.428	9.230
14	-6.826	-5.699	-3.330	-1.435	0.547	2.561	4.576	7.577	9.389
15	-6.725	-5.590	-3.203	-1.293	0.703	2.733	4.764	7.787	9.614
16	-6.595	-5.449	-3.039	-1.110	0.907	2.956	5.007	8.060	9.905
17	-6.435	-5.275	-2.836	-0.884	1.156	3.230	5.306	8.396	10.263
18	-6.247	-5.070	-2.597	-0.618	1.450	3.554	5.658	8.791	10.684
19	-6.035	-4.840	-2.329	-0.319	1.781	3.917	6.054	9.236	11.158
20	-5.805	-4.590	-2.037	0.006	2.141	4.312	6.485	9.719	11.672
21	-5.562	-4.326	-1.728	0.349	2.521	4.729	6.939	10.230	12.216
22	-5.311	-4.053	-1.410	0.703	2.913	5.159	7.408	10.756	12.776
23	-5.058	-3.778	-1.089	1.061	3.308	5.594	7.881	11.287	13.342
24	-4.807	-3.506	-0.772	1.414	3.699	6.023	8.349	11.812	13.901
25	-4.565	-3.243	-0.465	1.756	4.077	6.438	8.801	12.320	14.441
26	-4.335	-2.994	-0.174	2.080	4.435	6.831	9.229	12.800	14.953
27	-4.118	-2.758	0.100	2.386	4.774	7.203	9.634	13.255	15.437
28	-3.910	-2.533	0.363	2.679	5.098	7.558	10.021	13.689	15.899

29	-3.708	-2.315	0.617	2.962	5.411	7.902	10.396	14.110	16.347
30	-3.511	-2.101	0.867	3.240	5.719	8.240	10.764	14.522	16.786
31	-3.314	-1.888	1.115	3.516	6.025	8.575	11.130	14.933	17.224
32	-3.115	-1.673	1.366	3.795	6.334	8.915	11.499	15.347	17.665
33	-2.912	-1.453	1.622	4.081	6.650	9.262	11.878	15.772	18.118
34	-2.702	-1.225	1.888	4.377	6.978	9.622	12.270	16.212	18.587
35	-2.483	-0.987	2.165	4.686	7.320	9.997	12.679	16.671	19.077
36	-2.256	-0.741	2.452	5.005	7.673	10.385	13.102	17.145	19.583
37	-2.023	-0.488	2.747	5.334	8.037	10.785	13.538	17.634	20.105
38	-1.785	-0.229	3.050	5.670	8.410	11.195	13.984	18.135	20.639
39	-1.542	0.035	3.357	6.013	8.789	11.611	14.439	18.645	21.183
40	-1.296	0.303	3.669	6.360	9.173	12.034	14.899	19.162	21.735

D. Obese population

Gestational week	1%	2.3%	10%	25%	50%	75%	90%	97.7%	99%
5	-8.097	-6.805	-3.888	-1.435	1.171	3.827	6.523	10.60028	13.206
6	-8.253	-6.978	-4.101	-1.681	0.888	3.505	6.162	10.18046	12.748
7	-8.401	-7.142	-4.302	-1.914	0.621	3.202	5.822	9.78562	12.319
8	-8.538	-7.295	-4.489	-2.129	0.374	2.922	5.508	9.421545	11.923
9	-8.661	-7.432	-4.657	-2.323	0.152	2.671	5.228	9.096904	11.571
10	-8.766	-7.549	-4.800	-2.488	-0.037	2.458	4.990	8.820653	11.271
11	-8.849	-7.642	-4.915	-2.619	-0.187	2.288	4.801	8.601749	11.035
12	-8.908	-7.708	-4.995	-2.711	-0.293	2.170	4.670	8.449149	10.870
13	-8.938	-7.742	-5.036	-2.758	-0.346	2.110	4.603	8.371809	10.787
14	-8.935	-7.739	-5.033	-2.755	-0.342	2.114	4.608	8.378055	10.794
15	-8.901	-7.702	-4.987	-2.702	-0.282	2.182	4.684	8.465603	10.890
16	-8.840	-7.634	-4.904	-2.607	-0.173	2.304	4.820	8.623376	11.062
17	-8.756	-7.541	-4.791	-2.476	-0.024	2.472	5.007	8.840024	11.297
18	-8.653	-7.427	-4.652	-2.316	0.158	2.678	5.236	9.104202	11.584
19	-8.537	-7.297	-4.494	-2.134	0.366	2.911	5.495	9.404561	11.910
20	-8.411	-7.157	-4.322	-1.936	0.591	3.164	5.777	9.729753	12.263
21	-8.279	-7.010	-4.143	-1.730	0.826	3.428	6.071	10.06927	12.632
22	-8.143	-6.859	-3.959	-1.518	1.068	3.700	6.372	10.41775	13.010
23	-8.005	-6.706	-3.772	-1.302	1.314	3.976	6.679	10.77181	13.394
24	-7.866	-6.552	-3.584	-1.086	1.561	4.254	6.987	11.12807	13.781

25	-7.728	-6.398	-3.397	-0.870	1.807	4.530	7.295	11.48312	14.166
26	-7.592	-6.247	-3.212	-0.657	2.050	4.804	7.599	11.8336	14.545
27	-7.459	-6.100	-3.032	-0.450	2.286	5.070	7.895	12.17612	14.917
28	-7.329	-5.956	-2.857	-0.248	2.517	5.330	8.185	12.5099	15.278
29	-7.201	-5.814	-2.683	-0.048	2.745	5.588	8.471	12.84105	15.636
30	-7.071	-5.670	-2.507	0.155	2.977	5.849	8.762	13.17677	16.000
31	-6.936	-5.520	-2.325	0.365	3.216	6.120	9.064	13.52425	16.376
32	-6.794	-5.362	-2.132	0.588	3.470	6.405	9.382	13.8907	16.773
33	-6.640	-5.192	-1.925	0.827	3.742	6.711	9.723	14.28332	17.199
34	-6.473	-5.007	-1.699	1.087	4.038	7.044	10.093	14.70915	17.661
35	-6.292	-4.806	-1.454	1.369	4.359	7.404	10.493	15.16995	18.161
36	-6.099	-4.592	-1.192	1.670	4.702	7.787	10.920	15.66107	18.695
37	-5.896	-4.367	-0.917	1.985	5.061	8.190	11.368	16.17752	19.257
38	-5.686	-4.135	-0.633	2.312	5.434	8.608	11.834	16.71429	19.840
39	-5.471	-3.896	-0.342	2.647	5.815	9.037	12.312	17.26636	20.440
40	-5.254	-3.656	-0.048	2.986	6.202	9.473	12.799	17.82872	21.051

eTable 2. Range of gestational weight gain in kilograms to meet current guidelines (based on GWG curves based on the live-born population)

	Under weight		Normal weight		Over weight		Obese
Gestational week	Lower limit	Upper limit	Lower limit	Upper limit	Lower limit	Upper limit	Upper limit
5	0.983	2.945	-0.044	1.971	-0.456	1.736	0.227
6	0.975	2.935	-0.126	1.881	-0.671	1.496	-0.043
7	0.985	2.946	-0.186	1.814	-0.854	1.291	-0.298
8	1.018	2.983	-0.220	1.778	-1.002	1.127	-0.535
9	1.080	3.050	-0.222	1.775	-1.109	1.008	-0.746
10	1.175	3.153	-0.189	1.813	-1.172	0.938	-0.926
11	1.308	3.299	-0.114	1.896	-1.191	0.916	-1.068
12	1.485	3.492	0.008	2.029	-1.168	0.942	-1.166
13	1.711	3.739	0.179	2.217	-1.102	1.015	-1.214
14	1.986	4.040	0.399	2.459	-0.996	1.134	-1.208
15	2.305	4.389	0.665	2.752	-0.849	1.298	-1.152
16	2.665	4.783	0.975	3.093	-0.662	1.506	-1.053
17	3.061	5.215	1.328	3.480	-0.435	1.758	-0.916
18	3.486	5.681	1.719	3.911	-0.171	2.052	-0.747
19	3.937	6.174	2.146	4.380	0.126	2.382	-0.552
20	4.408	6.689	2.597	4.877	0.450	2.742	-0.337
21	4.889	7.215	3.059	5.386	0.793	3.124	-0.107
22	5.370	7.741	3.520	5.894	1.150	3.520	0.133
23	5.842	8.256	3.967	6.386	1.512	3.923	0.378
24	6.293	8.750	4.387	6.849	1.874	4.325	0.622
25	6.715	9.211	4.771	7.271	2.229	4.720	0.860
26	7.100	9.632	5.125	7.660	2.569	5.099	1.087
27	7.452	10.018	5.458	8.027	2.893	5.458	1.302
28	7.784	10.380	5.781	8.381	3.201	5.801	1.511
29	8.105	10.732	6.102	8.735	3.498	6.131	1.719
30	8.427	11.084	6.432	9.098	3.787	6.453	1.934
31	8.760	11.448	6.778	9.479	4.073	6.771	2.162
32	9.114	11.835	7.137	9.874	4.358	7.088	2.409
33	9.490	12.246	7.503	10.278	4.647	7.410	2.681
34	9.878	12.670	7.871	10.684	4.943	7.739	2.976
35	10.271	13.100	8.236	11.087	5.250	8.080	3.292

36	10.659	13.524	8.592	11.480	5.568	8.434	3.623
37	11.034	13.934	8.936	11.858	5.896	8.798	3.967
38	11.387	14.320	9.268	12.224	6.232	9.173	4.320
39	11.714	14.678	9.591	12.580	6.576	9.555	4.678
40	12.019	15.011	9.907	12.928	6.925	9.944	5.040

GWG, gestational weight gain.

eTable 3. Description of gestational weight gain by maternal characteristics; mean and standard deviation in low-risk population

	Number of woman	Gestational Weight Gain 0–15 weeks	16–27 weeks	28–37 weeks	38–43 weeks
**Number of data available**		16,734	17,334	17,443	17,669
**Pre-pregnancy BMI <18.5 kg/m²**	**3,018**	**0.9 (1.8)**	**5.5 (2.5)**	**9.0 (2.9)**	**11.1 (3.3)**
Maternal age, years					
≤25	586	1.2 (2.0)	5.9 (2.8)	9.7 (3.1)	12.1 (3.3)
26 to 34	1,877	0.8 (1.7)	5.5 (2.5)	8.9 (2.9)	10.9 (3.2)
≥35	555	0.8 (1.9)	5.4 (2.5)	8.6 (2.8)	10.5 (3.2)
Maternal height, cm					
≤155	672	0.8 (1.8)	5.3 (2.3)	8.7 (2.8)	10.7 (3.0)
156 to 161	1,435	0.9 (1.8)	5.6 (2.5)	9.0 (2.9)	11.1 (3.2)
≥162	911	0.9 (1.9)	5.6 (2.7)	9.3 (3.2)	11.3 (3.5)
Parity					
Multipara	1,933	0.8 (1.8)	5.5 (2.5)	9.0 (2.9)	11.0 (3.2)
Primipara	1,085	0.9 (1.9)	5.6 (2.6)	9.1 (3.0)	11.3 (3.4)
Smoking status					
Never smoke	1,740	0.6 (1.6)	5.1 (2.3)	8.5 (2.8)	10.5 (3.0)
Quit before realizing pregnancy	640	0.9 (1.8)	5.5 (2.4)	9.0 (2.7)	11.2 (3.0)
Quit after realizing pregnancy	416	1.7 (2.0)	6.9 (2.8)	10.6 (3.2)	12.9 (3.5)
Smoke during pregnancy	146	2.0 (2.0)	6.4 (2.9)	10.0 (3.2)	12.1 (3.8)
Missing	76	1.2 (1.7)	5.9 (2.5)	9.6 (3.1)	11.6 (3.9)
Previous diseases					
No	2,363	0.9 (1.8)	5.5 (2.5)	9.1 (2.9)	11.1 (3.2)
Yes	607	0.8 (1.8)	5.5 (2.5)	8.6 (3.0)	10.8 (3.3)
Missing	48	1.1 (2.1)	5.8 (2.6)	9.7 (2.8)	10.9 (3.8)
**Pre-pregnancy BMI 18.5–24.9 kg/m²**	**13,529**	**0.5 (2.0)**	**5.0 (2.8)**	**8.5 (3.3)**	**10.7 (3.5)**
Maternal age, years					
≤25	1,910	0.6 (2.1)	5.2 (3.0)	9.0 (3.5)	11.6 (3.8)
26 to 34	8,412	0.5 (2.0)	5.0 (2.8)	8.5 (3.2)	10.7 (3.4)
≥35	3,207	0.6 (2.1)	4.9 (2.7)	8.2 (3.1)	10.2 (3.4)
Maternal height, cm					
≤155	3,182	0.5 (2.0)	4.8 (2.7)	8.2 (3.2)	10.2 (3.4)
156 to 161	6,635	0.5 (2.0)	5.0 (2.8)	8.5 (3.2)	10.7 (3.5)
≥162	3,712	0.6 (2.1)	5.2 (2.9)	8.8 (3.3)	11.1 (3.6)
Parity					
Multipara	9,119	0.5 (2.0)	5.0 (2.8)	8.4 (3.2)	10.5 (3.4)
Primipara	4,410	0.6 (2.1)	5.1 (2.9)	8.7 (3.4)	11.0 (3.7)
Smoking status					
Never smoke	7,825	0.4 (1.9)	4.8 (2.7)	8.1 (3.1)	10.2 (3.3)
Quit before realizing pregnancy	3,271	0.5 (2.1)	5.0 (2.7)	8.5 (3.2)	10.7 (3.4)
Quit after realizing pregnancy	1,652	1.2 (2.2)	6.1 (3.1)	9.9 (3.6)	12.2 (3.9)
Smoke during pregnancy	534	1.5 (2.1)	5.9 (3.2)	9.5 (3.7)	11.9 (4.1)
Missing	247	0.6 (2.0)	5.5 (2.9)	9.4 (3.2)	11.8 (3.5)
Previous diseases					
No	10,438	0.6 (2.0)	5.0 (2.8)	8.5 (3.2)	10.7 (3.5)
Yes	2,948	0.5 (2.1)	4.9 (2.8)	8.3 (3.3)	10.5 (3.5)
Missing	143	0.7 (1.8)	5.5 (3.1)	9.2 (3.4)	11.5 (3.6)
**Pre-pregnancy BMI 25.0–29.9 kg/m²**	**1,153**	**0.2 (2.5)**	**3.3 (3.6)**	**6.5 (4.2)**	**8.6 (4.6)**
Maternal age, years					
≤25	177	0.5 (2.5)	4.1 (3.9)	7.6 (4.5)	9.7 (4.8)
26 to 34	688	0.1 (2.4)	3.2 (3.4)	6.3 (4.0)	8.5 (4.4)
≥35	288	0.1 (2.6)	3.3 (3.7)	6.1 (4.4)	8.0 (4.8)
Maternal height, cm					
≤155	282	0.4 (2.6)	3.5 (3.5)	6.5 (4.3)	8.6 (4.7)
156 to 161	575	0.2 (2.3)	3.4 (3.4)	6.6 (4.0)	8.7 (4.2)
≥162	296	0.0 (2.7)	3.2 (3.8)	6.3 (4.5)	8.4 (5.1)
Parity					
Multipara	869	0.2 (2.5)	3.2 (3.4)	6.4 (4.1)	8.4 (4.4)
Primipara	284	0.3 (2.6)	3.6 (3.9)	6.7 (4.6)	9.2 (5.0)

Smoking status					
Never smoke	582	-0.1 (2.3)	2.9 (3.4)	6.0 (4.1)	8.0 (4.3)
Quit before realizing pregnancy	319	0.1 (2.4)	3.3 (3.4)	6.5 (4.0)	8.5 (4.5)
Quit after realizing pregnancy	154	1.2 (2.8)	4.6 (4.3)	7.8 (4.8)	10.7 (5.4)
Smoke during pregnancy	63	0.7 (3.2)	3.9 (3.4)	7.1 (4.5)	8.4 (4.8)
Missing	35	0.5 (2.2)	3.9 (3.1)	7.6 (3.8)	9.2 (3.7)
Previous diseases					
No	858	0.2 (2.4)	3.4 (3.5)	6.6 (4.2)	8.6 (4.5)
Yes	280	0.2 (2.7)	3.1 (3.8)	6.2 (4.4)	8.5 (4.8)
Missing	15	-0.3 (1.5)	2.5 (2.2)	6.4 (3.6)	7.8 (3.2)
**Pre-pregnancy BMI ≥30.0 kg/m^2**	**250**	**-0.5 (2.7)**	**1.3 (3.8)**	**3.6 (4.3)**	**5.7 (4.9)**
Maternal age, years					
≤25	41	-0.2 (3.0)	1.0 (4.4)	3.8 (5.2)	5.2 (5.8)
26 to 34	149	-0.4 (2.6)	1.3 (3.5)	3.9 (3.8)	6.0 (4.5)
≥35	60	-0.7 (2.8)	1.6 (4.2)	2.9 (4.8)	5.3 (5.2)
Maternal height, cm					
≤155	62	0.0 (2.2)	2.2 (3.1)	4.9 (3.4)	6.0 (4.5)
156 to 161	110	-0.6 (3.0)	0.5 (3.6)	2.9 (4.3)	5.0 (4.4)
≥162	78	-0.6 (2.7)	1.7 (4.4)	3.7 (4.8)	6.4 (5.6)
Parity					
Multipara	195	-0.5 (2.8)	1.3 (3.8)	3.7 (4.3)	5.6 (4.9)
Primipara	55	-0.2 (2.4)	1.5 (3.7)	3.4 (4.2)	5.9 (4.7)
Smoking status					
Never smoke	109	-0.4 (2.5)	1.8 (3.7)	4.0 (4.1)	6.8 (4.3)
Quit before realizing pregnancy	65	-0.5 (2.7)	0.8 (3.4)	2.9 (4.1)	4.9 (4.3)
Quit after realizing pregnancy	36	-0.1 (3.0)	1.9 (4.4)	4.8 (5.3)	5.5 (6.9)
Smoke during pregnancy	25	-0.4 (2.5)	1.2 (3.2)	3.6 (3.5)	4.2 (4.2)
Missing	15	-2.3 (3.8)	-0.8 (4.7)	1.2 (4.1)	4.1 (5.4)
Previous diseases					
No	184	-0.4 (2.7)	1.6 (3.6)	4.1 (4.1)	6.0 (4.7)
Yes	59	-0.3 (2.4)	1.1 (3.9)	2.8 (4.4)	5.1 (5.1)
Missing	7	-3.7 (4.6)	-3.7 (4.5)	-0.5 (4.9)	1.5 (6.6)

BMI, body mass index.

eTable 4. Main effects of Bayesian mixed models (splines excluded) in low-risk population

	Estimate median	Exponential of Estimated median	Estimated Error	95% HDI	ESS
**Number of post-warmup samples (52,000)**	**Pre-pregnancy BMI <18.5 kg/m²**				
Maternal age, years					
≤25	0.015	1.015	0.006	[0.005 ; 0.026]	10,653
26 to 34	0.007	1.007	0.004	[-0.002 ; 0.015]	10,167
≥35	ref.				
Maternal height, cm					
≤155	-0.013	0.987	0.005	[-0.022 ; -0.004]	10,899
156 to 161	-0.005	0.995	0.004	[-0.012 ; 0.003]	10,347
≥162	ref.				
Parity					
Multipara	ref.				
Primipara	-0.003	0.997	0.003	[-0.009 ; 0.004]	10,735
Smoking status					
Never smoke	ref.				
Quit before realizing pregnancy	0.018	1.018	0.004	[0.01 ; 0.025]	10,758
Quit after realizing pregnancy	0.064	1.066	0.005	[0.055 ; 0.074]	10,631
Smoke during pregnancy	0.05	1.051	0.008	[0.035 ; 0.066]	11,058
Previous diseases					
No	ref.				
Yes	-0.008	0.992	0.004	[-0.016 ; 0]	11,098
sd(Intercept)	0.076		0.001	[0.073 ; 0.078]	17,214
**Number of post-warmup samples (12,000)**	**Pre-pregnancy BMI 18.5–24.9 kg/m²**				
Maternal age, years					
≤25	0.011	1.011	0.003	[0.005 ; 0.017]	13,723
26 to 34	0.003	1.003	0.002	[-0.001 ; 0.008]	13,580
≥35	ref.				
Maternal height, cm					
≤155	-0.020	0.980	0.003	[-0.025 ; -0.015]	12,240
156 to 161	-0.008	0.992	0.002	[-0.012 ; -0.003]	13,696
≥162	ref.				
Parity					
Multipara	ref.				
Primipara	0.005	1.005	0.002	[0.001 ; 0.009]	14,777
Smoking status					
Never smoke	ref.				
Quit before realizing pregnancy	0.012	1.012	0.002	[0.007 ; 0.016]	12,816
Quit after realizing pregnancy	0.055	1.057	0.003	[0.049 ; 0.06]	14,014
Smoke during pregnancy	0.053	1.054	0.005	[0.044 ; 0.063]	13,472
Previous diseases					
No	ref.				
Yes	-0.005	0. 995	0.002	[-0.009 ; -0.001]	15,539

sd(Intercept)	0.091		0.001	[0.09 ; 0.092]	18,978
**Number of post-warmup samples (60,000)**					
	**Pre-pregnancy BMI 25.0–29.9 kg/m²**				
Maternal age, years					
≤25	0.03	1.030	0.016	[-0.001 ; 0.061]	12,141
26 to 34	0.000	1.000	0.011	[-0.022 ; 0.022]	11,033
≥35	ref.				
Maternal height, cm					
≤155	0.011	1.011	0.013	[-0.016 ; 0.037]	11,718
156 to 161	0.010	1.010	0.011	[-0.012 ; 0.033]	10,879
≥162	ref.				
Parity					
Multipara	ref.				
Primipara	0.011	1.011	0.011	[-0.011 ; 0.033]	11,345
Smoking status					
Never smoke	ref.				
Quit before realizing pregnancy	0.015	1.015	0.011	[-0.007 ; 0.036]	10,649
Quit after realizing pregnancy	0.062	1.064	0.015	[0.033 ; 0.092]	11,311
Smoke during pregnancy	0.019	1.019	0.021	[-0.022 ; 0.059]	13,447
Previous diseases					
No	ref.				
Yes	-0.007	0.993	0.011	[-0.029 ; 0.014]	11,333
sd(Intercept)	0.137		0.004	[0.13 ; 0.144]	16,751
**Number of post-warmup samples (60,000)**	**Pre-pregnancy BMI ≥30.0 kg/m²**				
Maternal age, years					
≤25	0.022	1.022	0.038	[-0.052 ; 0.097]	14,917
26 to 34	0.051	1.052	0.030	[-0.007 ; 0.109]	12,782
≥35	ref.				
Maternal height, cm					
≤155	0.038	1.039	0.031	[-0.022 ; 0.099]	11,494
156 to 161	-0.052	0.949	0.028	[-0.107 ; 0.003]	12,954
≥162	ref.				
Parity					
Multipara	ref.				
Primipara	0.013	1.013	0.028	[-0.042 ; 0.068]	12,902
Smoking status					
Never smoke	ref.				
Quit before realizing pregnancy	-0.065	0.937	0.028	[-0.121 ; -0.01]	12,860
Quit after realizing pregnancy	0.011	1.011	0.034	[-0.055 ; 0.076]	14,746
Smoke during pregnancy	-0.024	0.976	0.04	[-0.102 ; 0.055]	15,178
Previous diseases					
No	ref.				
Yes	-0.014	0.986	0.026	[-0.065 ; 0.037]	14,189
sd(Intercept)	0.149		0.009	[0.133 ; 0.168]	20,758

BMI, body mass index; HDI, highest probability density (indicating which points of a distribution are most credible); ESS, Effective sample size (10,000 independent steps are recommended for an accurate 95% HDI)

Outcome of model is log-transformed weight gain (kg)

eTable 5. Range of gestational weight gain in kilograms to meet current guidelines (based on GWG curves based on the low risk population)

	Under weight		Normal weight		Over weight		Obese
Gestational week	Lower limit	Upper limit	Lower limit	Upper limit	Lower limit	Upper limit	Upper limit
5	0.831	2.785	-0.109	1.872	-0.421	1.762	-0.869
6	0.861	2.816	-0.175	1.800	-0.636	1.523	-0.906
7	0.899	2.857	-0.211	1.760	-0.820	1.317	-0.938
8	0.952	2.914	-0.213	1.758	-0.968	1.152	-0.962
9	1.025	2.994	-0.175	1.799	-1.075	1.033	-0.979
10	1.126	3.104	-0.096	1.885	-1.138	0.963	-0.985
11	1.263	3.254	0.023	2.016	-1.157	0.941	-0.979
12	1.442	3.450	0.182	2.191	-1.134	0.967	-0.958
13	1.670	3.699	0.379	2.407	-1.069	1.040	-0.921
14	1.948	4.002	0.612	2.664	-0.962	1.159	-0.865
15	2.270	4.355	0.882	2.960	-0.815	1.323	-0.789
16	2.635	4.753	1.185	3.294	-0.627	1.531	-0.689
17	3.037	5.193	1.522	3.664	-0.401	1.783	-0.566
18	3.473	5.669	1.891	4.069	-0.136	2.077	-0.419
19	3.939	6.179	2.286	4.504	0.162	2.409	-0.252
20	4.427	6.713	2.702	4.961	0.486	2.769	-0.067
21	4.925	7.258	3.132	5.435	0.830	3.151	0.134
22	5.422	7.802	3.571	5.917	1.187	3.548	0.347
23	5.905	8.330	4.012	6.402	1.550	3.951	0.572
24	6.363	8.831	4.448	6.882	1.913	4.354	0.805
25	6.784	9.292	4.875	7.351	2.268	4.749	1.045
26	7.167	9.711	5.285	7.801	2.610	5.129	1.291
27	7.521	10.098	5.675	8.230	2.934	5.489	1.541
28	7.856	10.464	6.046	8.639	3.243	5.832	1.797
29	8.182	10.820	6.401	9.029	3.540	6.162	2.058
30	8.509	11.178	6.743	9.405	3.831	6.485	2.322
31	8.848	11.548	7.073	9.768	4.117	6.803	2.590
32	9.203	11.936	7.395	10.122	4.403	7.121	2.862
33	9.569	12.336	7.710	10.469	4.692	7.442	3.136
34	9.940	12.742	8.021	10.811	4.989	7.772	3.413
35	10.309	13.145	8.331	11.151	5.296	8.113	3.693

36	10.670	13.540	8.640	11.491	5.615	8.467	3.974
37	11.017	13.920	8.948	11.830	5.944	8.833	4.256
38	11.346	14.280	9.256	12.169	6.281	9.207	4.540
39	11.659	14.623	9.564	12.507	6.625	9.590	4.824
40	11.959	14.952	9.871	12.845	6.975	9.979	5.109

GWG, gestational weight gain.
